# An Autopsy Case of CD4-Positive Lymphoproliferative Disorder at 38 Years Post-Transplantation Presenting With Cardiac Invasion and Cerebral Infarctions

**DOI:** 10.7759/cureus.94597

**Published:** 2025-10-14

**Authors:** Daisuke Hoshi, Yui Kusuno, Tomoko Takahashi, Hitoshi Yokoyama, Kengo Furuichi, Shin Ishizawa, Etsuko Kiyokawa

**Affiliations:** 1 Department of Oncologic pathology, Kanazawa Medical University, Ishikawa, JPN; 2 Department of Nephrology, Kanazawa Medical University, Ishikawa, JPN; 3 Department of Diagnostic and Therapeutic Radiology, Kanazawa Medical University, Ishikawa, JPN; 4 Department of Pathology, Toyama Prefectural Central Hospital, Toyama, JPN; 5 Department of Oncologic Pathology, Kanazawa Medical University, Ishikawa, JPN

**Keywords:** intravascular nk/t-cell lymphoma, post-transplant lymphoproliferative disorders, renal transplantation, t-cell lymphoma, thrombosis

## Abstract

Lymphoma is a rare but life-threatening complication following solid organ transplantation. The vast majority of these lymphomas arise from B cells associated with Epstein-Barr virus (EBV), and a small number of cases have a T-cell origin. We here report a rare post-transplantation autopsy case of progressive T-cell lymphoma with cardiac invasion and extensive intravascular dissemination, which became evident at 38 years following kidney transplantation.

The patient was a 72-year-old male who had received a kidney transplantation at 34 years of age. He had a 16-year history of recurrent cutaneous squamous cell carcinomas with lymph node metastases. Following hospitalization, he experienced dysuria and pancytopenia and subsequently developed dysarthria. Radiological examination revealed bilateral multiple cerebral infarctions. Despite supportive treatment, he died on the 31st hospital day. The autopsy revealed T-cell lymphoma, primarily originating from the retroperitoneal lymph node with cardiac invasion, causing systemic dissemination of tumor cells to small arteries and capillaries. These circulating tumor cells, which were negative for EBV, may have induced tumor embolization and cerebral infarction. This case was classified as monomorphic T/NK-cell post-transplant lymphoproliferative disorder and peripheral T-cell lymphoma, not otherwise specified, in the fourth and fifth World Health Organization classifications, respectively. Further research is required to clarify the association between immunosuppression and lymphoproliferative disorders.

## Introduction

Lymphomas occur in 1% of solid organ transplantation cases [1]. Since the first report published in 1968 [2], the term “post-transplant lymphoproliferative disorders (PTLDs)” has been used to describe these lymphomas. PTLDs are typically EBV-positive B-cell proliferations, consistent with the generally accepted theory that immune deficiency and dysregulation (IDD) induced by solid organ transplantation lead to EBV reactivation and subsequent lymphomagenesis. In contrast, T/NK-cell lymphomas are rare, and EBV is positive in a minority of cases [3-5]. Since the etiology of T/NK-cell PTLDs remains poorly understood [6,7], the latest version of the World Health Organization (WHO) classification regards T/NK-cell lymphoma as a coincidental disease following transplantation, except in the context of inborn errors of immunity [8].

Kidney transplantation is the most common solid organ transplantation. For patients with T/NK-cell PTLDs, 63%-69% had received kidney transplants [5,9], although the organ-specific incidence was the lowest [5]. In a meta-analysis from the French National PTLD Registry, among 500 recipients, 52 (10.4%) patients were diagnosed as T/NK-cell PTLD following kidney transplantation, and EBV was positive in 14/44 (31.8%) T/NK-cell cases and 104/178 (58.4%) B-cell cases [3]. Another report of T/NK-cell PTLD with various organ transplantations revealed that 28/81 (34.6%) of kidney transplantation cases and 46/136 (33.8%) of all tested cases were positive for EBV [5]. Compared with B-cell PTLD, T/NK-cell PTLD develops later; for example, the longest reported interval to onset is 324 months [5]. The 10-year survival rate after PTLD diagnosis is 49.8% for B-cell PTLD and 17.9% for T/NK-cell PTLD. Additionally, T/NK-cell PTLD outcomes are worse than those for T/NK-cell lymphomas unrelated to transplantation [3].

In Japan, an earlier study reported 12 cases of T/NK-cell PTLD among 24 cases of kidney transplantation [10]. EBV was positive in 83.3% of B-cell and 41.7% of T/NK-cell PTLDs. In a recent report, of the 1,973 kidney transplant recipients (two institutes overlapped in the previous report [10]), 241 (12.2%) developed various malignancies, including 37 PTLD cases (1.9% of kidney transplantation) [11]. EBV status is not described in this study.

Most types of lymphoma cells are located in the extravascular spaces, while intravascular lymphoma cells are predominantly in the vasculature. However, in limited cases, non-intravascular lymphoma cells, which invade the heart, could disseminate through the blood vessels, eventually leading to tumor embolization [12-14].

We here experienced a rare autopsy case with the following three characteristics: (1) T-cell lymphoma progressively developed at 38 years following kidney transplantation, which, to the best of our knowledge, represents the longest interval; (2) negative for viral infection: EBV by in situ hybridization and human T-lymphotropic virus type 1 (HTLV-1) by serology test; and (3) the lymphoma showed cardiac muscle invasion, followed by intravascular dissemination, leading to cerebral infarctions.

## Case presentation

A 72-year-old male patient underwent living kidney transplantation from his sister 38 years before his death and was treated with azathioprine, cyclosporine, and methylprednisolone. He had a 16-year history of multiple recurring cutaneous squamous cell carcinomas (SCCs) of the fingers, head, neck, and chest, which were sequentially resected (51 surgeries overall). Six months before hospitalization, the patient noticed a mass in the left cervical region, which was surgically resected after one month. The specimen was diagnosed as an SCC metastasis. At this time, the physician noted left oculomotor nerve palsy and suspected Tolosa-Hunt syndrome; however, contrast-enhanced magnetic resonance imaging (MRI) did not reveal any mass around the cavernous sinus. Three weeks before hospitalization, another cutaneous SCC in the anterior neck region was resected. On the same day, positron emission tomography/computed tomography (PET/CT) was performed to investigate for SCC metastasis. PET/CT revealed abnormal uptake in the left upper cervical lymph node and the retroperitoneal region in front of the left kidney; both were suspicious of neoplastic origin. The left upper cervical lymph node revealed SCC metastasis as examined by cytological examination one week later, whereas the retroperitoneal region in front of the left kidney was not pathologically investigated.

**Figure 1:**
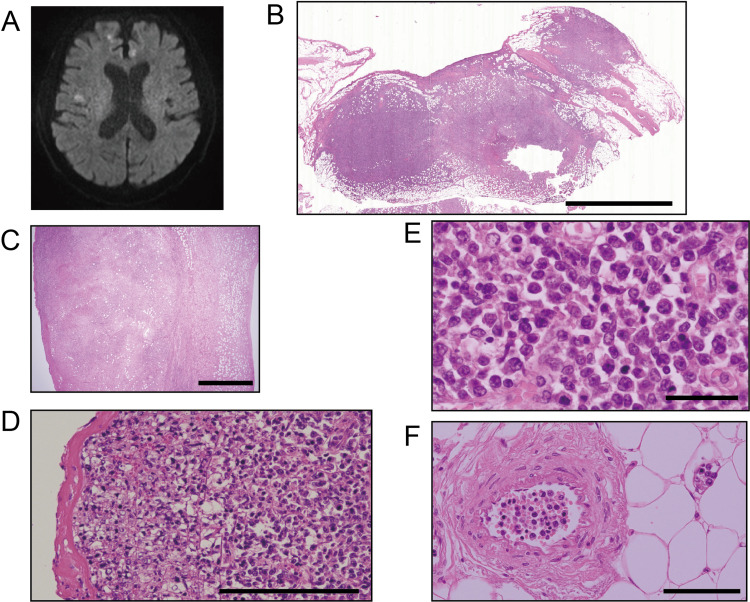
Cerebral infarctions and extension of the lymphoma (A) Diffusion-weighted magnetic resonance imaging. Infarcted areas are shown as white nodules; (B–F) hematoxylin and eosin (H&E) staining of the lymphoma and its extension; (B) retroperitoneal mass; (C) the right atrial wall with full-thickness invasion; (D) lymphoma cells invading the luminal surface of the right atrium with fibrin deposition; (E) high-power view; and (F) lymphoma cells in the small arteries and capillaries. Scale bars: (B) 5 mm, (C) 2 mm, (D) 200 µm, (E) 40 µm, and (F) 100 µm.

The patient was hospitalized for cervical lymph node resection; however, he subsequently developed dysuria, leg pain, and pancytopenia. The surgery was postponed, and he received symptomatic treatment. On the 17th hospital day, he developed dysarthria, and an MRI revealed numerous bilateral cerebral infarcts (Figure 1A). On the 31st hospital day, the patient died without a definitive diagnosis due to progressive pancytopenia. With the consent of his family, an autopsy was performed to determine the diagnosis. Brain autopsy was not allowed.

Various organs revealed several small nodules, each measuring several centimeters. The largest ones were detected in the heart and retroperitoneal spaces, including the perirenal region (Figure 1B). The cardiac wall exhibited multiple masses encompassed by atypical lymphoid cells, with full-thickness invasion noted in the right atrium (Figure 1C). This invasion led to the exposure of the mass to the luminal surface, leading to fibrin deposition (Figure 1D). In addition, atypical cells formed thrombi with fibrin in the lumen of the left ventricle. These findings suggest that tumor thrombi could circulate systemically. Furthermore, tumor masses were detected in the lungs, pleura, stomach, colon, mesentery, liver, gallbladder, bladder, prostate, and testis. These masses comprised sheet-like structures of atypical lymphoid cells ranging from centroblastoid to pleomorphic forms, which invaded adipose tissue with mild fibrosis (Figure 1E). Additionally, tumor invasion induced pericarditis with 265 mL of pericardial fluid.

All examined organs, except for the spleen and bone marrow, contained these atypical cells in their blood vessels, including small arteries and capillaries (Figure 1F), whereas the bone marrow demonstrated significant hemophagocytosis. Atypical intravascular cells were not present in the surgical specimen obtained before death, but were noted in the cervical SCC specimen obtained during autopsy.

**Figure 2:**
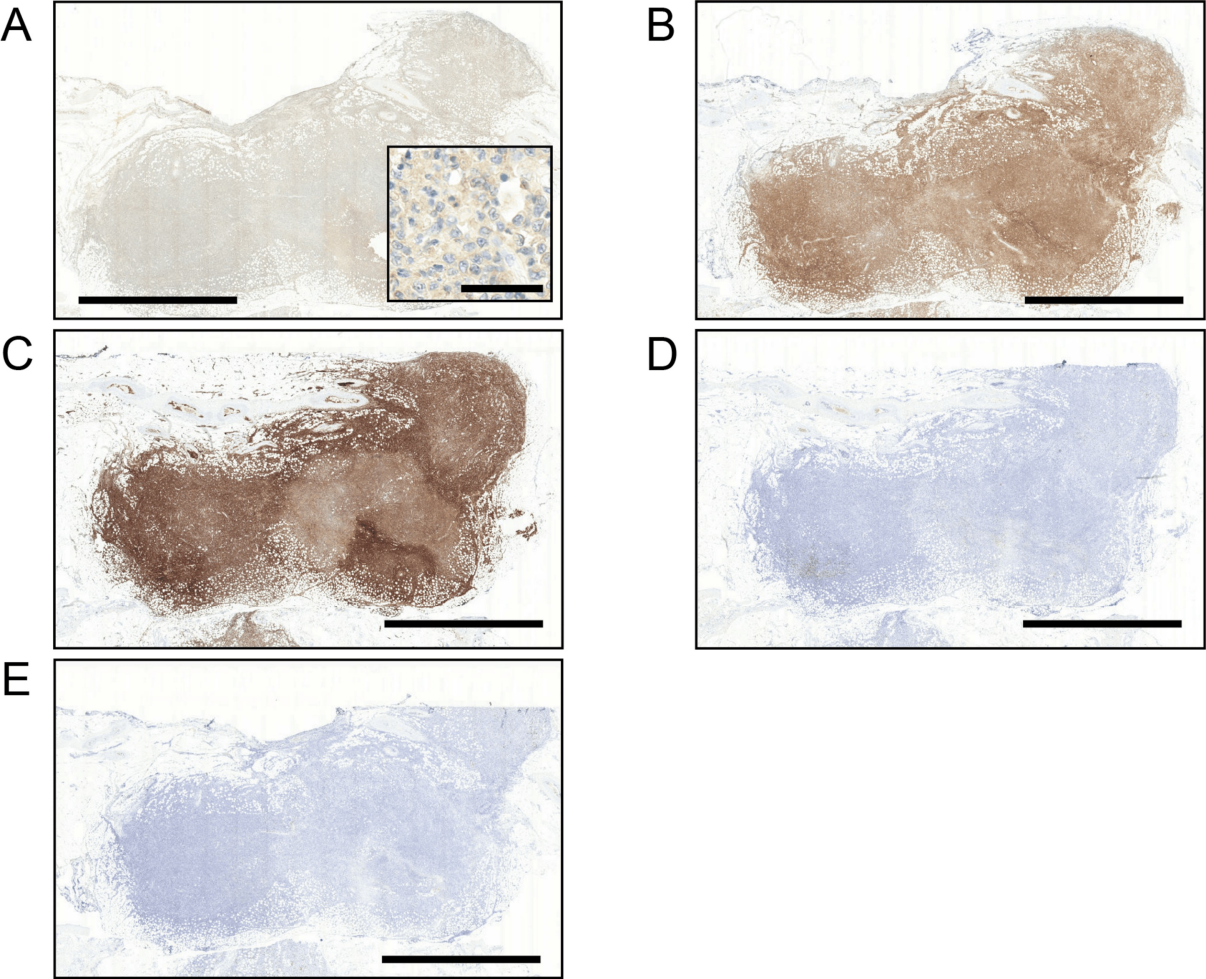
Representative immunohistochemistry of the retroperitoneal mass. (A) CD3, (B) CD4, (C) CD5, (D) CD7, and (E) CD8. Scale bars: 5 mm, 50 µm for the inset in (A).

Immunostaining revealed that these atypical lymphoid cells exhibited features characteristic of helper T cells, as they were positive for CD3, CD4, and CD5, and negative for CD7, CD8, CD20, CD56, myeloperoxidase, and lysozyme (Figures 2A-2E, Table 1). These cells were negative for CD21, and no CD21-positive dendritic cell network enlargement was observed. CD45 was negative in the tumor cells within the mass area but positive in those within the vasculature. BCL6, CD10, and PD1 were negative in the atypical cells. EBV-encoded small RNA in situ hybridization was negative in the atypical cells, and no significant increase was observed in the surrounding cells. Antibody testing using the serum cryopreserved approximately three weeks before his death was negative for HTLV-1 infection.

**Table 1:** Summary of immunohistochemistry and in situ hybridization CD3: Cluster of Differentiation 3, CD4: Cluster of Differentiation 4, CD5: Cluster of Differentiation 5, CD45: Custer of Differentiation 45, ALK: Anaplastic Lymphoma Kinase, BCL6: B-cell Lymphoma 6, CD7: Cluster of Differentiation 7, CD8: Cluster of Differentiation 8, CD10: Cluster of Differentiation 10, CD20: Cluster of Differentiation 20, CD21: Cluster of Differentiation 21, CD30: Cluster of Differentiation 30, CD56: Cluster of Differentiation 56, CD79a: Cluster of Differentiation 79a, PD-1: Programmed Cell Death Protein 1, EBER: Epstein–Barr Virus Encoded RNA

Marker	Tumor cells	Surrounding cells
CD3	Positive	
CD4	Positive	
CD5	Positive	
CD45	Positive (intravascular), Negative (mass)	
ALK	Negative	
BCL6	Negative	
CD7	Negative	
CD8	Negative	
CD10	Negative	
CD20	Negative	
CD21	Negative	No overgrowth
CD30	Negative	
CD56	Negative	
CD79a	Negative	
Myeloperoxidase	Negative	
Lysozyme	Negative	
PD-1	Negative (only a few positive)	
EBER	Negative	No increase

As differential diagnoses, we considered peripheral T-cell lymphoma, not otherwise specified (PTCL-NOS), given the helper T-cell phenotype and multiple nodules throughout the body, and intravascular NK/T-cell lymphoma (IVNKTL), due to the phenotype and abundant intravascular tumor cells. However, we excluded IVNKTL for the following reasons. First, typical intravascular lymphoma cells are confined to capillaries [8], whereas in this case, tumor cells were observed not only within capillaries but also in arteries, accompanied by cardiac wall invasion with tumor thrombi. Second, CD4-positive IVNKTL is extremely rare [15], and even when positive, the intensity is weak [16]. In our case, CD4 was clearly positive (Figure 2B). Therefore, we concluded that the accumulation of tumor cells within blood vessels was due to secondary dissemination following cardiac invasion by the tumor. Based on these findings, the final diagnosis was PTCL-NOS.

The cause of death was circulatory failure resulting from tumor invasion of the heart, pericardial fluid accumulation, and loss of blood cells due to hemophagocytosis.

## Discussion

Following the fourth and fifth WHO classifications, this case was diagnosed as monomorphic T/NK-cell PTLD and PTCL-NOS, respectively. This inconsistency arises from different categorizations of lymphomas in immunocompromised conditions. The fourth edition included B- and T/NK-cell lymphomas in lymphomas arising in immunocompromised states, including post-transplantation [17]. In contrast, the fifth edition introduced the category “lymphoid proliferations and lymphomas associated with IDD” only within the B-cell lymphoma section [8]. This alteration underscores the ambiguity of the association between T-cell lymphomas and IDD. A study has indicated that precursor lesions and EBV infection are observed in B-cell proliferations and lymphomas in IDD. In contrast, T-cell lymphomas in IDD show no obvious precursors and are frequently negative for EBV [6].

Our patient had undergone a renal transplantation, had been treated with immunosuppressive drugs, and developed multiple cutaneous SCCs. Despite these immunocompromised conditions, his lymphoma exhibited EBV negativity, suggesting that lymphoma and immunodeficiency can be coincidental. However, the causal relationship between T-cell lymphoma and immunodeficiency is not completely denied because some EBV-negative lymphomas, like hepatosplenic T-cell lymphoma, are clearly associated with IDD [18]. To date, as different mutation patterns between T-cell PTLD with or without EBV have not been identified [19], the development of T-cell PTLD might be independent of EBV.

This case exhibited a fulminant course, which fits the previous finding that systemic T/NK-cell PTLDs exhibited a worse prognosis than peripheral T-cell lymphomas of non-transplant patients [3]. Although we did not histologically examine the brain, massive cardiac invasion and tumor thrombi in the left ventricle suggest that cerebral infarctions were caused by tumor embolization, as seen in several reports indicating an association between tumor embolization and cardiac lesions [12-14].

The primary site of the patient’s lymphoma could be a lymph node near the left kidney, identified in autopsy, since abnormal uptake of PET/CT was observed in the same area. At the time of the PET/CT, SCC metastasis was one of the differential diagnoses; however, the possibility of an inflammatory mass or a tumor other than SCC was also considered. Although imaging did not reveal a significant mass, the possibility that this tumor may have caused the oculomotor nerve palsy six months before admission could not be completely ruled out. Steroid diabetes could be another possible cause of the palsy.

## Conclusions

This case demonstrates that lymphomas can develop as a late complication even 38 years after solid organ transplantation. Notably, this lymphoma was an aggressive T-cell lymphoma involving cardiac infiltration and cerebral infarction. While the latest WHO classification only cautiously addresses the association between T/NK-cell lymphomas and organ transplantation, epidemiological data suggest differences in prognosis between transplant recipients and the general population. In this case, there were clear signs of immunodeficiency, as exemplified by the development of skin cancer, further supporting the need for a more in-depth investigation into the relationship between solid organ transplantation and T/NK-cell lymphomas. Additionally, as illustrated in this case, the diagnosis of lymphoma may not be established during the patient’s lifetime. Therefore, physicians must maintain a high index of suspicion for lymphoma in post-transplant patients.
